# Perioperative treatment and biomarker analysis of LP002, an anti‐PD‐L1 antibody, plus chemotherapy in resectable gastric and gastroesophageal junction cancer

**DOI:** 10.1002/cam4.5414

**Published:** 2022-11-07

**Authors:** Jia‐lin Tang, Bo Zhang, Jian‐ping Xu, Ling Qi, Dao Xin, Lin Wang, Bing‐zhi Wang, Yan‐tao Tian, Yong Li, Jing Huang

**Affiliations:** ^1^ Department of Medical Oncology, National Cancer Center/National Clinical Research Center for Cancer/Cancer Hospital Chinese Academy of Medical Sciences and Peking Union Medical College Beijing China; ^2^ Department of Pathology and Resident Training Base, National Cancer Center/National Clinical Research Center for Cancer/Cancer Hospital Chinese Academy of Medical Sciences and Peking Union Medical College Beijing China; ^3^ Department of Pancreatic and Gastric Surgery, National Cancer Center/National Clinical Research Center for Cancer/Cancer Hospital Chinese Academy of Medical Sciences and Peking Union Medical College Beijing China; ^4^ Department of Thoracic Surgery, National Cancer Center/National Clinical Research Center for Cancer/Cancer Hospital Chinese Academy of Medical Sciences and Peking Union Medical College Beijing China; ^5^ National Cancer Center/National Clinical Research Center for Cancer/Cancer Hospital & Shenzhen Hospital Chinese Academy of Medical Sciences and Peking Union Medical College Shenzhen China

**Keywords:** gastric cancer, gastroesophageal junction cancer, histopathological regression, immunotherapy, perioperative treatment

## Abstract

**Background:**

The addition of immune checkpoint inhibitors to perioperative chemotherapy in operable gastric or gastroesophageal junction (GEJ) cancer has become one of the research hotspots, while reliable biomarkers for efficacy are lacking. We conducted a phase 1 trial to assess the safety and efficacy of LP002, an anti‐PD‐L1 antibody, plus chemotherapy as perioperative treatment in patients with gastric or GEJ cancer.

**Methods:**

We enrolled patients with resectable and PD‐L1 positive gastric or GEJ cancers. Eligible patients received three preoperative and six postoperative cycles of intravenous LP002 with cisplatin and 5‐fluorouracil, repeated every 2 weeks. The primary endpoint was safety. Secondary endpoints included rate of margin‐free (R0) resection and pathological complete response (pCR). We also characterized changes in the tumor immune microenvironment using multiplex immunofluorescence (MIF) staining and next‐generation sequencing (NGS) with pre‐ and post‐treatment tumor samples.

**Results:**

Thirty patients were enrolled, of whom 28 had GEJ cancer. With a median follow‐up of 7.9 months, all patients completed preoperative treatment, and 27 patients underwent surgery. Twenty‐four patients underwent R0 resection. Six patients (20.0%) had Mandard tumor regression grade (TRG) 1–3, including one achieving pCR. Twenty‐seven patients had treatment‐related adverse events (TRAEs), while grade 3–4 TRAEs were observed in 11 patients. No treatment‐related deaths occurred. MIF staining revealed that TRG 1–3 group was associated with a higher density of PD‐L1+/CD68+ cells in the pre‐treatment tumor parenchyma than TRG 4–5 group (*p* = 0.048). NGS studies with paired pre‐ and post‐treatment tumor samples revealed the disappearance of pre‐existing mutations, the emergence of new mutations, and variations in the abundance of mutations after preoperative LP002 and chemotherapy. Meanwhile, tumor mutational burden decreased in patients with TRG 1–3 (*p* = 0.0313).

**Conclusions:**

LP002 plus cisplatin and 5‐fluorouracil are safe in patients with gastric or GEJ cancer, and patient selection via appropriate biomarkers is needed in the future.

## Graphical abstract

In this study, perioperative treatment with PD‐L1 blockade plus chemotherapy provided clinical benefits for patients with resectable gastric or gastroesophageal junction cancer, with tolerable toxicities. NGS studies performed with paired pre‐ and post‐treatment tumor samples showed disappearance of pre‐existing mutations, emergence of new mutations, and variations in the abundance of mutations after preoperative LP002 and chemotherapy. In addition, tumor mutational burden decreased after preoperative treatment in the TRG 1‐3 group.

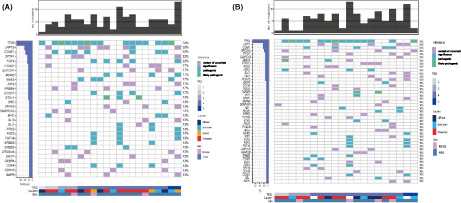

## 1 INTRODUCTION

Gastric cancer is among the most common malignancies worldwide, ranking fifth in incidence and fourth in mortality globally.^1^ The past 15 years have witnessed major advances in the multimodal treatment of locally advanced, resectable diseases. Several randomized clinical trials have demonstrated the survival benefits of the addition of perioperative chemotherapy to surgery alone^2^, ^3^, ^4^, ^5^ and have thus established this strategy as one of the standard treatments for patients with resectable gastric and gastroesophageal junction (GEJ) cancers. Specifically, cisplatin plus 5‐fluorouracil (5‐FU) improved overall survival (OS), disease‐free survival (DFS), and curative resection rates compared to surgery in a randomized phase 3 trial^3^ and has since become a common regimen used in the perioperative setting.

In recent years, based on the growing evidence of the clinical activity of immune checkpoint inhibitors (ICI) in metastatic gastric or GEJ cancer,^6^, ^7^, ^8^ there has also been great interest in adding ICIs to neoadjuvant or perioperative chemotherapy for treating patients with locally advanced, resectable disease. Currently, reports on the outcomes of neoadjuvant or perioperative ICI plus chemotherapy in gastric or GEJ cancer are few and limited to early‐phase trials.^9^, ^10^, ^11^, ^12^, ^13^, ^14^, ^15^ The preliminary results from these trials seemed promising, with margin‐free (R0) resection rates between 97% and 100% and pathological complete response (pCR) rates between 10% and 33%.^9^, ^10^, ^11^, ^12^, ^13^, ^14^, ^15^ However, a number of important questions remain unanswered, including the optimal choice of ICI and chemotherapy backbone and the impact of preoperative ICI plus chemotherapy on the tumor immune microenvironment. More importantly, although PD‐L1 expression has been recognized as a potential biomarker to select patients who may derive greater benefit from PD‐1 blockade in metastatic gastric or GEJ cancer,^7^, ^8^, ^16^, ^17^ its predictive role for the resectable disease has not been established, and reliable biomarkers for response and survival are lacking for these patients.

LP002 is a humanized, anti‐PD‐L1 monoclonal antibody. In a dose‐escalation study, patients with gastric adenocarcinoma and esophageal squamous cell carcinoma responded to single‐agent LP002. We included an expansion cohort in the phase I trial to evaluate the safety and efficacy of LP002 plus cisplatin and 5‐FU as a perioperative treatment for patients with PD‐L1 positive, locally advanced, resectable gastric or GEJ cancer. To identify potential biomarkers for pathological responses, we also investigated pre‐ and post‐treatment genetic alterations of the tumor and changes in the tumor microenvironment. Here, we present the short‐term outcomes of this cohort, including safety, margin‐free (R0) resection rate, pathological responses to this novel combination, and results of biomarker analysis.

## 2 PATIENTS AND METHODS

### 2.1 Patients

In this prospective, open‐label, single‐arm phase 1 study, we enrolled patients aged between 18 and 75 years who were treatment‐naïve and had histologically confirmed, locally advanced (cT2‐4a, any N, M0) resectable gastric or type 1 to 3 GEJ cancer with positive PD‐L1 expression. Other eligibility criteria included an Eastern Cooperative Oncology Group performance status of 0 or 1 and adequate bone marrow and organ functions. We excluded patients who had the active autoimmune disease or required chronic use of steroids or immunosuppressive drugs, had uncontrolled intercurrent conditions including active infection, and severe cardiac or gastrointestinal diseases.

The trial was conducted in accordance with the Declaration of Helsinki and Good Clinical Practice guidelines. The research protocol was reviewed and approved by our institutional review board. All patients provided written informed consent before the study. The trial was registered at ClinicalTrials.gov (NCT04755543).

### 2.2 Procedures

Eligible patients received three preoperative and six postoperative cycles of intravenous LP002 (Taizhou Houde Aoke Technology Co., Ltd.) at a fixed dose of 900 mg on day 1, intravenous cisplatin (Jiangsu Hansoh Pharmaceutical Group Co., Ltd.) at 50 mg/m^2^ on day 1, and continuous infusion of 5‐FU (Tianjin Kingyork Pharmaceuticals Co., Ltd.) at 2000 mg/m^2^ for 48 h from day 1. All treatment cycles were repeated every 2 weeks. Interruptions, delays, or discontinuations of certain drugs for specific toxicities were permitted at the discretion of the investigators. Dose reductions were allowed for cisplatin and 5‐FU, if appropriate; however, the doses for LP002 could not be modified.

Surgery was scheduled for 4–6 weeks after the completion of the third cycle of preoperative treatment. Postoperative treatment was initiated 4 weeks after surgery.

### 2.3 Assessments

PD‐L1 expression in the tumor was assessed centrally by immunohistochemistry (IHC) with the 6E8 antibody (Shuwen Biotech Co. Ltd.). PD‐L1 expression in the tumor cells (TC) and the tumor‐infiltrating immune cells (IC) were reported separately. PD‐L1 TC expression was defined as the number of tumor cells with positive PD‐L1 membranous staining divided by the total number of tumor cells ×100%. IC expression was defined as the number of PD‐L1‐stained tumor‐infiltrating immune cells divided by the total number of tumor‐infiltrating immune cells ×100%. For patient screening, PD‐L1 positivity was defined as TC≥1% or IC≥1%.

Baseline tumor imaging via computed tomography (CT) or magnetic resonance imaging (MRI) was performed before preoperative treatment, every 6 weeks during pre‐ and postoperative treatment, and every 12 weeks thereafter. Imaging studies were also performed before surgical resection to confirm resectability and before the first cycle of postoperative treatment.

Laboratory tests, including complete blood counts, chemistry, urinalysis, thyroid function tests, and electrocardiograms, were performed before treatment initiation and then repeated every 2 weeks. The adverse events (AEs) overserved in the study were graded according to the National Cancer Institute Common Terminology Criteria for Adverse Events, version 4.03. Patients were followed up every 3 months after the completion of postoperative treatment until death.

The resected specimens were processed according to local standard procedures and reviewed by experienced, independent pathologists. Pathological staging, including primary site (T) and regional lymph nodes (N), was performed and recorded according to the 8th edition of the American Joint Committee on Cancer (AJCC) classification. Pathological regression in the primary tumor and lymph nodes was assessed and reported using the Mandard tumor regression grading system.^18^

### 2.4 Multiplex immunofluorescence staining and sequencing analysis

We performed multiple immunofluorescence (MIF) staining and 733‐gene‐based next‐generation sequencing (NGS) on pre‐treatment biopsy samples and post‐treatment surgical samples. Using MIF staining, we evaluated the expression of PD‐L1, CD4, CD8, CD56, PD‐1, FoxP3, CD56, CD20, CD68, and CD163 on tumor and/or tumor‐infiltrating immune cells, as well as the presence of tertiary lymphoid structures (TLS). PD‐L1 expression by MIF was reported with combined positive score (CPS), defined as the number of PD‐L1‐positive cells (including tumor cells, lymphocytes, and macrophages) divided by the total number of tumor cells × 100. Using NGS, we evaluated the tumor mutational burden (TMB) and somatic and germline alterations. Details regarding MIF staining and NGS are provided in the Supplementary Data (Supplementary Methods).

### 2.5 Outcomes

The primary endpoint was safety. Secondary endpoints included the proportion of patients with margin‐free (R0) resection, proportion of patients with pathological complete response (ypT0N0 according to the 8th edition of the AJCC classification), disease‐free survival (DFS), and overall survival (OS). DFS was defined as the time from the start of the preoperative treatment to disease progression, relapse, or death from any cause. OS was defined as the time from the start of the preoperative treatment to death from any cause. The exploratory endpoints were the pre‐ and post‐treatment dynamics of genetic alterations or markers in the tumor immune microenvironment, and their correlation with the pathological response to the study treatment.

### 2.6 Statistical analysis

Analyses of the primary and secondary endpoints were performed on an intention‐to‐treat basis in all patients who had received at least one dose of any of the study drugs, regardless of whether they underwent surgery. The sample size of 30 patients was determined based on the exploratory nature of this study. Safety and efficacy data are presented as the frequency and percentage of patients involved. Time‐to‐event variables were estimated using the Kaplan–Meier method in SPSS (version 26). Statistics of density in immune cells between Mandard tumor regression grade (TRG) 1–3 and TRG 4–5 were analyzed using the Mann–Whitney U test. Immune cell density and TMB statistics in pre‐ and post‐treatment paired samples were compared using the Wilcoxon matched‐pairs signed‐rank test. All reported *p*‐values were based on two‐tailed testing, and statistical significance was set at *p* < 0.05. Statistical analyses of biomarkers were performed using GraphPad Prism software 8.3.0 (GraphPad Software, Inc.).

## 3 RESULTS

### 3.1 Patient characteristics

Thirty eligible patients were enrolled between September 2, 2020, and July 16, 2021 (Table 1). The median age was 64.5 years (range: 50–74 years), and 26 patients (86.7%) were men. Most of the primary tumors were located in the GEJ (*N* = 28, 93.3%). Twenty‐nine patients (96.7%) had adenocarcinoma and one had a small cell neuroendocrine tumor. The baseline clinical tumor stage was cT3/T4a in 28 patients (93.3%) and the clinical node stage was N+ in 26 patients (78.3%).

**TABLE 1:** Baseline characteristics of the 30 enrolled patients

Characteristic	*N* (%)
Age (years)	
≤60	4 (13.3)
61–75	26 (86.7)
Gender	
Male	26 (86.7)
Female	4 (13.3)
ECOG	
0	27 (90.0)
1	3 (10.0)
Tumor location	
GEJ Siewert type 1	1 (3.3)
GEJ Siewert type 2	21 (70.0)
GEJ Siewert type 3	6 (20.0)
Stomach	2 (6.7)
Pathological type	
Adenocarcinoma	29 (96.7)
Small cell neuroendocrine tumor	1 (3.3)
cT‐stage^a^	
T2	2 (6.7)
T3	23 (76.6)
T4a	5 (16.7)
cN‐stage^a^	
N0	1 (3.3)
N+	26 (86.7)
Unclear	3 (10.0)
Lauren's type	
Diffuse	6 (20.0)
Intestinal	15 (50.0)
Mixed	4 (13.3)
Not evaluable according to Lauren	1 (3.4)
Missing	4 (13.3)
PD‐L1 TC expression	
0%	23 (76.6)
≥1%	7 (23.4)
PD‐L1 IC expression	
0%	3 (10.0)
≥1%	27 (90.0)
MSI and/or MMR status	
MSS and/or MMR‐proficient	26 (86.7)
Unknown	4 (13.3)

Abbreviations: GEJ, gastroesophageal junction; IC, immune cell; MMR, mismatch repair; MSI, microsatellite instability; MSS, microsatellite stable; TC, tumor cell.

^a^ Clinical tumor stage and clinical nodal (cN) stage were assessed using endoscopic ultrasound or CT/PET‐CT and classified according to the eighth edition of the AJCC classification.

### 3.2 Treatment and surgical outcomes

As of the data cutoff date (November 1, 2021), the median follow‐up was 7.9 months (range: 5.1–10.6 months). All patients completed the planned preoperative treatment, and 27 patients (90.0%) underwent surgery, with a median time from the start of preoperative treatment to surgery of 80 days (range: 55–111 days). Among them, 15 (55.6%) experienced a delay in surgery due to the COVID‐19 pandemic. The reasons for surgery not being performed were metastatic disease confirmed before surgery in one patient and patient request in two cases. Postoperative treatment was initiated in 25 patients, and the reason for the two patients not starting postoperative treatment was patients request. Three patients did not complete all six postoperative treatment cycles because of adverse events. Postoperative treatment was ongoing in 10 patients as of the data cutoff.

Twenty‐four patients (80.0%) underwent R0 resection, while three underwent R1 resection. One patient (3.3%) with GEJ adenocarcinoma had Mandard TRG 1 (ypT0) and tumor‐free lymph nodes (ypN0) and therefore achieved a pathological complete response. Six patients (20.0%) achieved TRG 1–3 (Table 2). At the time of data cut‐off, one patient had disease relapse 3 months after the completion of perioperative treatment, one patient who did not undergo surgery died of disease progression, while the other patients had no disease progression, relapse, or death, and the survival data were not mature.

**TABLE 2:** Results of pathological evaluation

Pathological evaluation	*N* (%)
TNM stage after operation	
ypT0N0	1 (3.7)
Stage I	3 (11.1)
Stage II	7 (25.9)
Stage III	16 (59.3)
Pathology T‐stage	
ypT0	1 (3.7)
ypT2	4 (14.8)
ypT3	13 (48.1)
ypT4	9 (33.3)
Pathology *N*‐stage	
ypN0	4 (14.8)
ypN1	8 (29.6)
ypN2	9 (33.3)
ypN3	6 (22.2)
Pathological regression (as per the Mandard criteria)	
TRG 1	1 (3.7)
TRG 2	2 (7.4)
TRG 3	3 (11.1)
TRG 4	19 (70.4)
TRG 5	2 (7.4)

### 3.3 Safety

Twenty‐seven patients experienced at least one TRAE during perioperative treatment (Table 3). Most TRAEs were grade 1 to 2. The most common grade 3 toxicities were nausea (23.3%), decreased neutrophil count (16.7%), and anorexia (6.7%). Eleven patients had immune‐related AEs (irAEs), mostly grade 1 hyperthyroidism, whereas no grade 5 irAEs occurred during the study. Surgical complications, including anastomotic leakage, anemia, pneumonia, and abdominal distention related to surgery were observed in three patients. Pre‐ and postoperative dose modifications were performed in two and 12 patients, respectively. The most common reasons for dose modification were nausea and anorexia. No treatment‐related deaths occurred during this period.

**TABLE 3:** Treatment‐related adverse events

	Grade 1 *N* (%)	Grade 2 *N* (%)	Grade 3 *N* (%)	Grade 4 *N* (%)
Hematologic toxicity				
Anemia	13 (43.3)	8 (26.7)	0	0
Neutrophil count decreased	5 (16.7)	7 (23.3)	5 (16.7)	0
Platelet count decreased	4 (13.3)	2 (6.7)	0	0
White blood cell decreased	3 (10.0)	10 (33.3)	0	0
Non‐hematologic toxicity				
Hyperthyroidism^a^	7 (23.3)	0	0	0
Nausea	6 (20.0)	9 (30.0)	7 (23.3)	0
Anorexia	6 (20.0)	5 (16.7)	2 (6.7)	0
Vomiting	6 (20.0)	3 (10.0)	1 (3.3)	0
Fatigue	6 (20.0)	1 (3.3)	0	0
ALT increased	3 (10.0)	0	0	0
AST increased	3 (10.0)	0	0	0
Creatinine increased	3 (10.0)	0	0	0
Hypothyroidism^a^	1 (3.3)	1 (3.3)	0	0
Paroxysmal atrial tachycardia^a^	1 (3.3)	1 (3.3)	0	0
Allergic reaction	0	2 (6.7)	0	0
Proteinuria	0	1 (3.3)	0	0
Oral ulceration	0	1 (3.3)	0	0
Acute kidney injury^a^	0	0	0	1 (3.3)

Abbreviations: ALT, alanine aminotransferase; AST, aspartate aminotransferase.

^a^ These events were attributed to LP002 or LP002 and chemotherapy and were categorized as immune‐related adverse events.

### 3.4 Biomarker analyses

**FIGURE 1:**
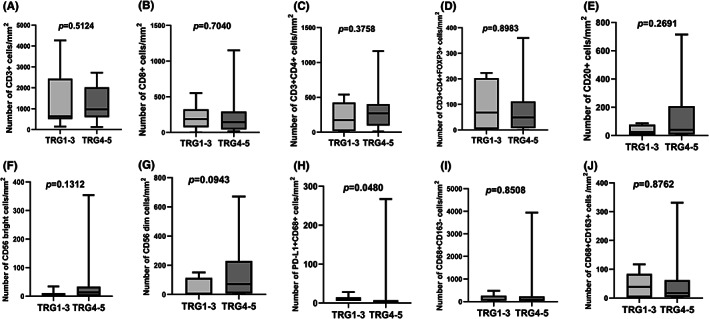
Immune cell infiltration in pre‐treatment tumor parenchyma by multiple immunofluorescence staining. Densities of the following cells are compared between patients with TRG 1–3 (*N* = 6) and TRG 4–5 (*N* = 21): (A) infiltrating CD3+ cells, (B) infiltrating CD8+ cells, (C) infiltrating CD3 + CD4+ cells, (D) infiltrating CD3 + CD4 + FOXP3+ cells, (E) infiltrating CD20+ cells, (F) infiltrating CD56 bright cells, (G) infiltrating CD56 dim cells, (H) infiltrating PD‐L1 + CD68+ cells, (I) infiltrating CD68 + CD163‐ cells, and (J) infiltrating CD68 + CD163+ cells.

Paired pre‐ and post‐treatment samples from 27 patients were analyzed using MIF staining. PD‐L1 expression by MIF staining showed that three pre‐treatment samples had CPS≥1 (range: 1–5), whereas the other 24 samples had CPS <1. We divided the patients into two groups according to pathological responses: TGR 1–3 representing marked tumor regression, and TRG 4–5 showing mild or no regression. In the pre‐treatment samples, we found that the TRG 1–3 group had a higher density of PD‐L1/CD68 double‐positive cells in the tumor parenchyma than the TRG 4–5 group (*p* = 0.048) (Figure 1). Other pre‐treatment biomarkers, including CD3+, CD8+, CD3 + CD4+, CD20+, CD68+ tumor‐associated macrophages (TAM), NK cells, and TLS, were not different between the TRG 1–3 and TRG 4–5 groups. Assessment of post‐treatment changes revealed a decrease in CD20+ B cells and CD68 + TAM cell infiltration in both tumor parenchyma and stroma compared with paired pre‐treatment samples in the TRG 4–5 group, but not in the TRG 1–3. The full results of MIF staining in both the tumor parenchyma and stroma are provided in the Supplementary Data (Figure S1–S6).

**FIGURE 2:**
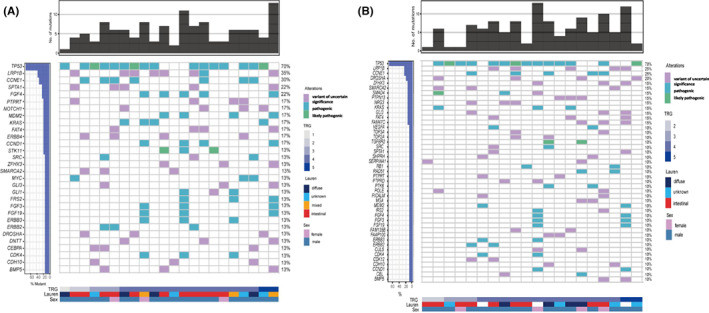
Genetic alterations in (A) 23 pre‐treatment tumor samples, and (B) 20 post‐treatment tumor samples. Each column represents one patient sample.

**FIGURE 3:**
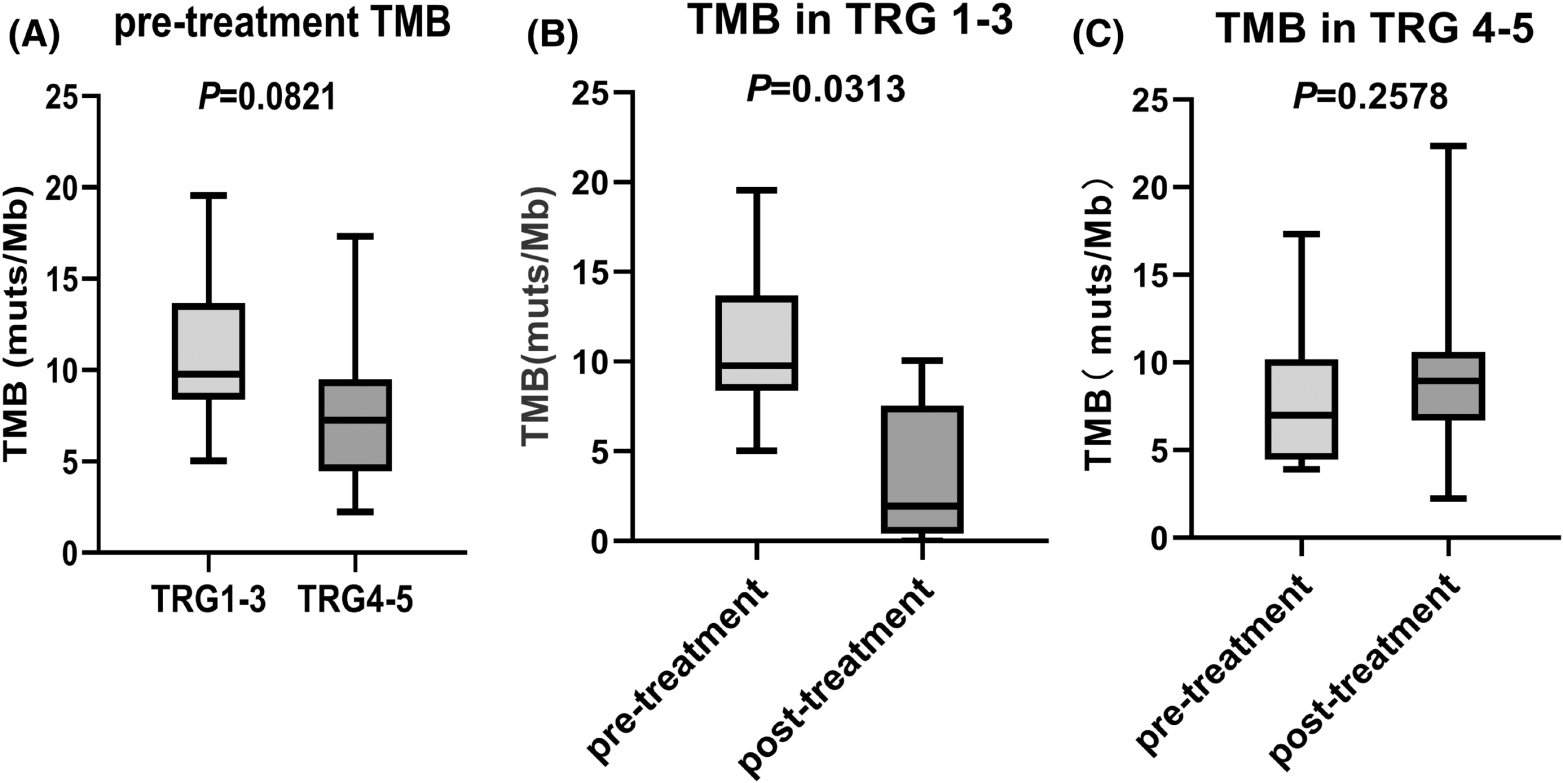
Analyses of the tumor mutational burden (TMB). (A) TMB in pre‐treatment samples in patients with Mandard tumor regression grade (TRG) 1–3 (*N* = 6) versus TRG 4–5 (*N* = 17) . (B) Pre‐ and post‐treatment TMB in patients with TRG 1–3 (*N* = 6). TMB of the patient with complete pathological regression (TRG 1) was calculated as 0 muts/Mb. (C) Pre‐ and post‐treatment TMB in patients with TRG 4–5 (*N* = 14).

The 733‐gene‐based NGS was performed with pre‐treatment samples from 23 patients and post‐treatment samples from 20 patients. The most frequently mutated genes were TP53 (70%), LRP1B (35%), CCNE1 (30%), and FGF4 (22%) (Figure 2). By comparing paired pre‐ and post‐treatment sequencing results, we observed the disappearance of pre‐existing mutations, the emergence of new mutations, and variations in the abundance of mutations, suggesting that preoperative LP002 and chemotherapy may alter the genetic alterations in the tumor. We found that TMB levels in the TRG 1–3 group decreased after preoperative treatment (*p* = 0.0313) (Figure 3), suggesting that the reduction in TMB at the molecular level was consistent with pathological remission.

Evaluation of mismatch repair (MMR) protein expression was evaluated in 19 patients, all of whom were MMR‐proficient. Meanwhile, all 23 patients with pre‐treatment samples in our exploratory NGS analyses had microsatellite‐stable (MSS) tumors. Altogether, we found that 26 patients were either MMR‐proficient or MSS, whereas the MMR/MSI status of the other four patients was unknown.

## 4 DISCUSSION

In the present study, we evaluated perioperative PD‐L1 blockade combined with chemotherapy in patients with resectable gastric or GEJ cancer. Perioperative LP002 plus cisplatin and 5‐fluorouracil had a manageable safety profile. Meanwhile, 80.0% of the patients underwent R0 resection, and 20.0% of the patients showed remarkable tumor regression (Mandard TRG 1–3). To our knowledge, this is the first study to demonstrate pre‐ and post‐treatment changes in genomic alterations and immune cell infiltration in resectable gastric or GEJ cancer patients treated with an immune checkpoint inhibitor plus chemotherapy.

Several studies have provided evidence that tumor regression correlates with survival in patients with esophagogastric cancers^19^, ^20^; therefore, it is frequently chosen as a surrogate endpoint in clinical trials involving neoadjuvant treatments. The percentage of patients achieving pCR after preoperative LP002 and chemotherapy was small in our present trial (3.3%) compared with the reference data in historical trials. The reported rates of pT0 or pCR after preoperative chemotherapy from previous phase III studies for gastric cancer patients were 2.75–16%.^2^, ^3^, ^4^, ^5^ In recent phase II trials with the addition of anti‐PD‐1 antibodies to chemotherapy as preoperative treatment, the pCR rates ranged from 10 to 33.3%.^11^, ^12^, ^13^, ^14^, ^15^

Several reasons might explain the limited proportion of patients with pCR in the current study. First, although we selected patients with PD‐L1 positive tumors, the evaluation of PD‐L1 expression was different from that for CPS. Previous clinical studies in patients with advanced gastric or GEJ cancer receiving ICI alone or ICI plus chemotherapy suggested that PD‐L1 CPS might be a potential predictive biomarker of efficacy.^7^, ^8^, ^16^, ^17^ One phase II study evaluating neoadjuvant anti‐PD‐1 antibody plus chemotherapy in patients with resectable gastric or GEJ cancer reported a pCR rate of 19.4%, and the percentage of patients with baseline tumor PD‐L1 CPS >1 in this report was 58.3%.^15^ In contrast, only three pre‐treatment samples (10.0%) in our present trial showed PD‐L1 CPS >1 by MIF staining. In other words, most enrolled patients were PD‐L1 negative in terms of CPS, which might have limited the benefit from the addition of LP002. Our results suggest that patient selection via appropriate biomarkers is important in perioperative PD‐L1 inhibition and chemotherapy for resectable gastric or GEJ cancer patients, and the PD‐L1 CPS might still be a useful predictive tool for response in this setting. Gastric cancer is a kind of significant heterogeneous disease. In order to improve the accuracy of detecting PD‐L1 protein expression, it is necessary to select the most representative area in the tumor sample and do multiple biopsies. Second, the impact of two‐drug cytotoxic regimens on tumor regression could be different from that of three‐drug regimens. Although there were no head‐to‐head comparisons between the two‐ and three‐drug regimens in perioperative chemotherapy for gastric or GEJ cancer, evidence exists for metastatic disease. The addition of docetaxel to 5‐FU and oxaliplatin improved the response rate (48.6% vs. 28.17%) and the proportion of patients having complete response (5.6% vs. 0%) in the first‐line treatment of locally advanced or metastatic esophagogastric adenocarcinoma in a randomized phase II study.^21^ Likewise, the reported pCR rates after preoperative three‐drug chemotherapies were also numerically higher. In the FNCLCC‐FFCD study, three of the 109 patients (2.75%) in the chemotherapy plus surgery group achieved pT0 after preoperative cisplatin and 5‐FU.^3^ In another phase III trial using oxaliplatin and S‐1 (SOX) as perioperative chemotherapy for patients with locally advanced gastric or GEJ cancer, 5.6% of patients in the perioperative‐SOX group were staged ypT0N0 after preoperative treatment.^5^ In comparison, preoperative chemotherapy with docetaxel, oxaliplatin, and 5‐FU (FLOT) or epirubicin, cisplatin, and 5‐FU or capecitabine (ECF/ECX) resulted in pCR rates of 16% and 6%, respectively, in the phase III FLOT trial.^4^ Moreover, the dosage of 5‐FU in the FLOT trial was 2600 mg/m^2^ delivered as a 24‐h infusion, compared with a lower intensity of 5‐FU at 2000 mg/m^2^ over 48 h in our study, especially on the first day. Despite the limitations of cross‐trial comparisons, the effect of chemotherapy intensity on histologic tumor regression should not be overlooked. Third, surgery delay due to the COVID‐19 epidemic might have a negative impact on our outcomes since the median time from the first treatment to surgery was 80 days, which exceeds the time window of 58–72 days specified in the protocol.

The safety profile observed for LP002 plus chemotherapy was similar to the known safety profiles of individual treatments, with no new safety signals identified. The most common TRAEs in our study were hematologic and gastrointestinal chemotherapy‐related toxicities. Immune‐related adverse events were predominantly grade 1 or 2 thyroid dysfunctions. Since ≥ grade 3 kidney injuries were rare in previous studies with anti‐PD‐L1 antibodies,^22^, ^23^, ^24^ the case of acute grade 4 kidney injury in our study was probably also associated with the nephrotoxic effect of cisplatin in addition to the suspected immune‐mediated etiology.

The MIF staining results of the tumor immune microenvironment in our study suggest that the baseline infiltration density of PD‐L1/CD68 double‐positive macrophages might be a predictive indicator of the efficacy of LP002 plus chemotherapy. A previous study found that high PD‐L1 expression in macrophages in patients with non‐small cell lung cancer treated with PD‐1/PD‐L1 inhibitors was associated with better OS.^25^ Based on these results, the role of baseline density of PD‐L1/CD68 double‐positive macrophages as a predictive biomarker for PD‐1/PD‐L1 blockade warrants further verification in studies with a larger sample size. Our study also revealed that by comparing the pre‐ and post‐treatment MIF staining results, the infiltration density of CD20+ B cells and CD68+ macrophages decreased after preoperative treatment in patients with TRG 4–5. Whether reduced infiltration of CD20+ B cells and CD68+ TAM is associated with poor prognosis requires further collection of survival data.

The limitations of our study include the small number of enrolled patients, the methodology to define patients' PD‐L1 positivity, and the absence of a control group of standard perioperative chemotherapy. Additionally, the follow‐up time was short in this report, as the survival outcomes were not mature. DFS and OS for these patients are currently under follow‐up and will be updated when available.

## 5 CONCLUSIONS

Perioperative LP002 with cisplatin and 5‐fluorouracil showed manageable safety and clinical benefits in patients with resectable gastric or GEJ cancer. Predictive biomarkers for pathological response and survival and the optimal chemotherapy regimen to accompany PD‐L1 blockade should be the focus of future studies while precision medicine is awaited.

## AUTHOR CONTRIBUTIONS

**Jialin Tang:** Data curation (equal); formal analysis (equal); resources (equal); software (equal); validation (equal); writing – original draft (lead); writing – review and editing (equal). **Bo Zhang:** Data curation (equal); formal analysis (equal); resources (equal); software (equal); validation (equal); writing – original draft (lead); writing – review and editing (equal). **Jianping Xu:** Data curation (equal); formal analysis (equal); resources (equal); software (equal); validation (equal); writing – review and editing (equal). **Ling Qi:** Resources (equal); validation (equal); visualization (equal); writing – review and editing (equal). **Dao Xin:** Resources (equal); software (equal); validation (equal); writing – review and editing (equal). **lin wang:** Resources (equal); validation (equal); writing – review and editing (equal). **Bingzhi Wang:** Resources (equal); validation (equal); writing – review and editing (equal). **Yantao Tian:** Conceptualization (equal); data curation (equal); resources (equal); supervision (equal); validation (equal); writing – review and editing (equal). **Yong Li:** Conceptualization (equal); investigation (equal); project administration (equal); resources (equal); supervision (equal); writing – review and editing (equal). **Jing Huang:** Conceptualization (lead); data curation (lead); formal analysis (lead); investigation (lead); project administration (lead); resources (lead); supervision (lead); validation (lead); visualization (lead); writing – original draft (supporting); writing – review and editing (lead).

## FUNDING INFORMATION

This study was supported by Taizhou Houde Aoke Technology Co., Ltd., and the Sanming Project of Medicine in Shenzhen (No. SZSM201612085).

## CONFLICT OF INTEREST

The authors declare no potential conflicts of interest.

## CLINICAL TRIAL REGISTRATION

The trial was registered at ClinicalTrials.gov (NCT04755543).

## ETHICS APPROVAL STATEMENT

The research protocol was reviewed and approved by the institutional review board of the Cancer Hospital, Chinese Academy of Medical Sciences. The ID for ethics approval is 19–051/1836.

## PATIENT CONSENT STATEMENT

All patients provided written informed consent before the study.

## Supporting information


Appendix S1

Appendix S2

## Data Availability

The data that support the findings in this study are accessible from the corresponding author on reasonable request and permission from the study sponsor.
