# Supplementary material for: Perioperative treatment and biomarker analysis of LP002, an anti‐PD‐L1 antibody, plus chemotherapy in resectable gastric and gastroesophageal junction cancer
Source: Cancer Med. 2022 Nov 7;12(5):5639–48. doi: 10.1002/cam4.5414 (PMC10028060; doi:10.1002/cam4.5414)
Supplement: Supplementary file 1 — Appendix S1 [file CAM4-12-5639-s002.docx]

**Supplementary Data**

**Supplementary - Methods**

**Tissue processing and genomic DNA extraction**

Formalin-fixed paraffin-embedded (FFPE) tissue sections were evaluated for tumor cell content using hematoxylin and eosin (H&E) staining. Only samples with a tumor content of ≥20% were eligible for subsequent analyses. FFPE tissue sections were placed in a 1.5 microcentrifuge tube and deparaffinized with mineral oil. Samples were incubated with lysis buffer and proteinase K at 56 °C overnight until the tissue was completely digested. The lysate was subsequently incubated at 80 °C for 4 hours to reverse formaldehyde crosslinks. Genomic DNA was isolated from tissue samples using the ReliaPrep™ FFPE gDNA Miniprep System (Promega) and quantified using the Qubit™ dsDNA HS Assay Kit (Thermo Fisher Scientific) following the manufacturer’s instructions.

**Library preparation and targeted capture**

DNA extracts (30-200 ng) were sheared to 250 bp fragments using an S220 focused-ultrasonicator (Covaris). Libraries were prepared using the KAPA Hyper Prep Kit (KAPA Biosystems) following the manufacturer’s protocol. The concentration and size distribution of each library were determined using a Qubit 3.0 fluorometer (Thermo Fisher Scientific) and a LabChip GX Touch HT Analyzer (PerkinElmer) respectively.

For targeted capture, indexed libraries were subjected to probe-based hybridization with a customized NGS panel targeting 733 cancer-related genes and 500 selected MSI loci. Repetitive elements were filtered out from intronic baits according to the annotation by UCSC Genome RepeatMasker ^1^. The xGen® Hybridization and Wash Kit (IDT) was employed for hybridization enrichment. Briefly, 500 ng indexed DNA libraries were pooled to obtain a total amount of 2 μg of DNA. The pooled DNA sample was then mixed with human cot DNA and xGen Universal Blockers-TS Mix and dried down in a SpeedVac system. The Hybridization Master Mix was added to the samples and incubated in a thermal cycler at 95℃ for 10 min, before being mixed and incubated with 4 μl of probes at 65℃ overnight. The target regions were captured following the manufacturer’s instructions. The concentration and fragment size distribution of the final library were determined using a Qubit 3.0 fluorometer (Thermo Fisher Scientific) and a LabChip GX Touch HT Analyzer (PerkinElmer) respectively.

**DNA sequencing, data processing, and variant calling**

The captured libraries were loaded onto a NovaSeq 6000 platform (Illumina) for 100 bp paired-end sequencing with a mean sequencing depth of 989.Raw data of paired samples (an FFPE sample and its normal control) were mapped to the reference human genome hg19 using the Burrows-Wheeler Aligner (v0.7.12) ^2^. PCR duplicate reads were removed and sequence metrics were collected using Picard (v1.130) and SAMtools (v1.1.19), respectively. Variant calling was performed only in the targeted regions. Somatic single nucleotide variants (SNVs) were detected using an in-house developed R package to execute a variant detection model based on binomial test. Local realignment was performed to detect indels. Variants were then filtered by their unique supporting read depth, strand bias, base quality as previously described ^3^. All variants were then filtered using an automated false positive filtering pipeline to ensure sensitivity and specificity at an allele frequency (AF) of ≥ 5%. Single-nucleotide polymorphism (SNPs) and indels were annotated by ANNOVAR against the following databases: dbSNP (v138), 1000Genome and ESP6500 (population frequency > 0.015). Only missense, stopgain, frameshift and non-frameshift indel mutations were kept. Copy number variations (CNVs) and gene rearrangements were detected as described previously ^3^.

**TMB**

For data processing and reads mapping, please refer to “DNA sequencing, data processing, and variant calling ”. TMB was defined as the number of nonsynonymous and synonymous somatic SNVs and indels in examined coding regions, with driver mutations excluded. All SNVs and indels in the coding region of targeted genes, including missense, silent, stop gain, stop loss, in-frame and frameshift mutations, were considered.

**Tumor immune microenvironment (TIME) by multiplex immunofluorescence (MIF)**

Multiplex immunofluorescence staining was conducted using the Akoya OPAL Polaris 7-Color Automation IHC kit (NEL871001KT). FFPE tissue slides were first deparaffinized in a BOND RX system (Leica Biosystems) and then incubated sequentially with primary antibodies targeting CD163 (Abcam, ab182422, 1:500), CD68 (Abcam, ab213363, 1:1000), PD-1 (CST, D4W2J, 86163S, 1:200), PD-L1 (CST, E1L3N, 13684S, 1:400)， CD3 (Dako, A0452, 1:1), CD4 (Abcam, ab133616, 1:100), CD8 (Abcam, ab178089, 1:200), CD56 (Abcam, ab75813, 1:1000), CD20 (Dako, L26, IR604, 1:1), FOXP3 (Abcam, ab20034, 1:100) and pan-CK ( Abcam, ab7753, 1:100) (Akoya Biosciences). This was followed by incubation with secondary antibodies and corresponding reactive Opal fluorophores. Nuclei acids were stained with DAPI. Tissue slides that were bound with primary and secondary antibodies but not fluorophores were included as negative controls to assess autofluorescence. Multiplex stained slides were scanned using a Vectra Polaris Quantitative Pathology Imaging System (Akoya Biosciences) at 20 nm wavelength intervals from 440 nm to 780 nm with a fixed exposure time and an absolute magnification of ×200. All scans for each slide were then superimposed to obtain a single image. Multilayer images were imported to inForm v.2.4.8 (Akoya Biosciences) for quantitative image analysis. Tumor parenchyma and stroma were differentiated by Pan-CK staining. The quantities of various cell populations were expressed as the number of stained cells per square millimeter and as the percentage of positively stained cells in all nucleated cells.

**References:**

1. Karolchik D, Hinrichs A S, Furey T S, et al. The UCSC Table Browser data retrieval tool[J]. Nucleic Acids Res, 2004,32(Database issue):D493-D496.

2. Li H, Durbin R. Fast and accurate short read alignment with Burrows-Wheeler transform[J]. Bioinformatics, 2009,25(14):1754-1760.

3. Su D, Zhang D, Chen K, et al. High performance of targeted next generation sequencing on variance detection in clinical tumor specimens in comparison with current conventional methods[J]. J Exp Clin Cancer Res, 2017,36(1):121.
