# Supplementary material for: Perioperative treatment and biomarker analysis of LP002, an anti‐PD‐L1 antibody, plus chemotherapy in resectable gastric and gastroesophageal junction cancer
Source: Cancer Med. 2022 Nov 7;12(5):5639–48. doi: 10.1002/cam4.5414 (PMC10028060; doi:10.1002/cam4.5414)
Supplement: Supplementary file 2 — Appendix S2 [file CAM4-12-5639-s001.pdf]

Supplementary Figures

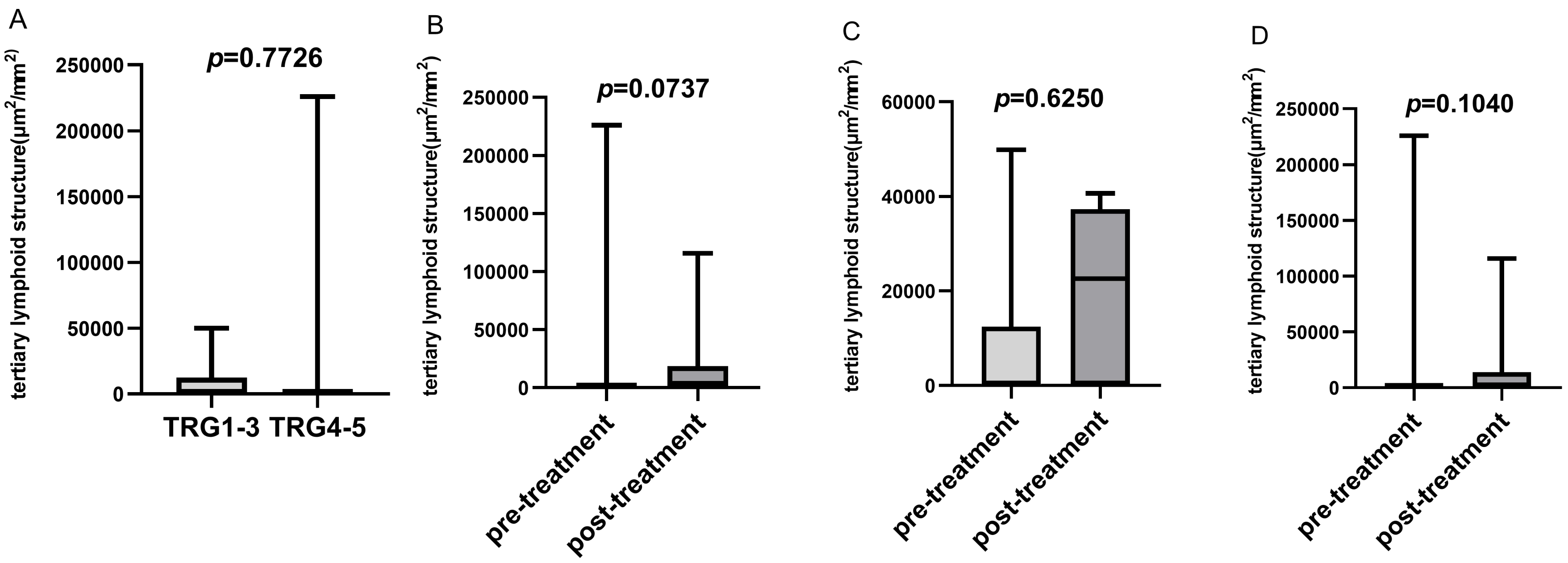

Figure S1: Analyses of tertiary lymphoid structures in region of tumor parenchyma and stroma by multiple immunofluorescence staining. (A) Tertiary lymphoid structures in pre-treatment samples in patients with Mandard tumor regression grade (TRG) 1-3 (N=6) versus TRG 4-5 (N=21). (B) Tertiary lymphoid structures in pre- and post-treatment samples (N=27). (C) Pre- and post-treatment tertiary lymphoid structures in patients with TRG 1-3 (N=6). (D) Pre- and post-treatment tertiary lymphoid structures in patients with TRG 4-5 (N=21).

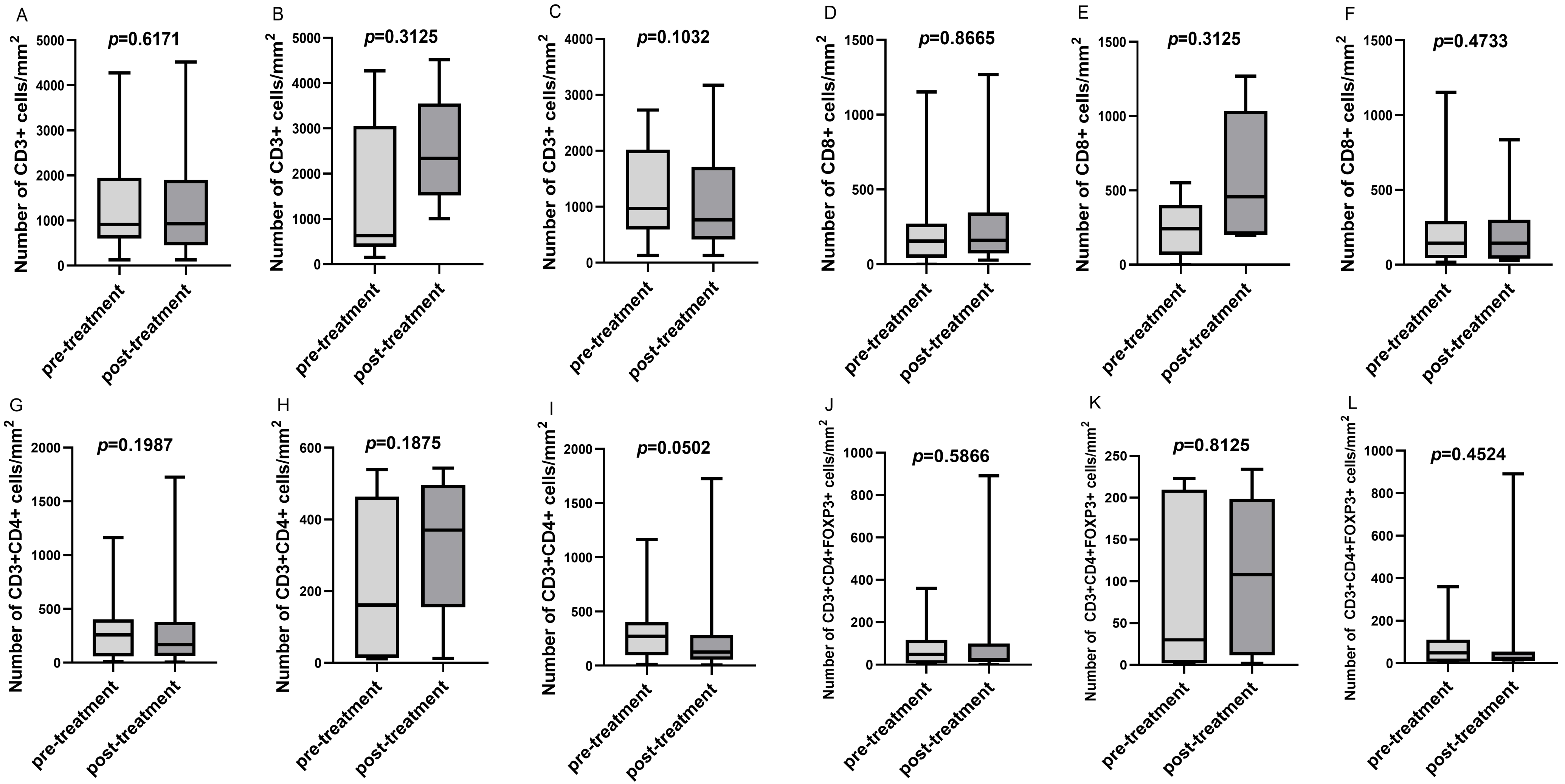

Figure S2: Pre- and post-treatment immune cell infiltration in tumor parenchyma by multiple immunofluorescence staining. Densities of the following cells are compared between pre- and post-treatment samples: (A)infiltrating CD3+ cells (N=26), (B) infiltrating CD3+ cells in patients with TRG 2-3 (N=5), (C) infiltrating CD3+ cells in patients with TRG 4-5 (N=21), (D) infiltrating CD8+ cells (N=26), (E) infiltrating CD8+ cells in patients with TRG 2-3 (N=5), (F) infiltrating CD8+ cells in patients with TRG 4-5 (N=21), (G) infiltrating CD3+CD4+ cells (N=26), (H) infiltrating CD3+CD4+ cells in patients with TRG 2-3 (N=5), (I) infiltrating CD3+CD4+ cells in patients with TRG 4-5 (N=21), (J) infiltrating CD3+CD4+FOXP3+ cells (N=26), (K) infiltrating CD3+CD4+FOXP3+ cells in patients with TRG 2-3 (N=5), and (L) infiltrating CD3+CD4+FOXP3+ cells in patients with TRG 4-5 (N=21).

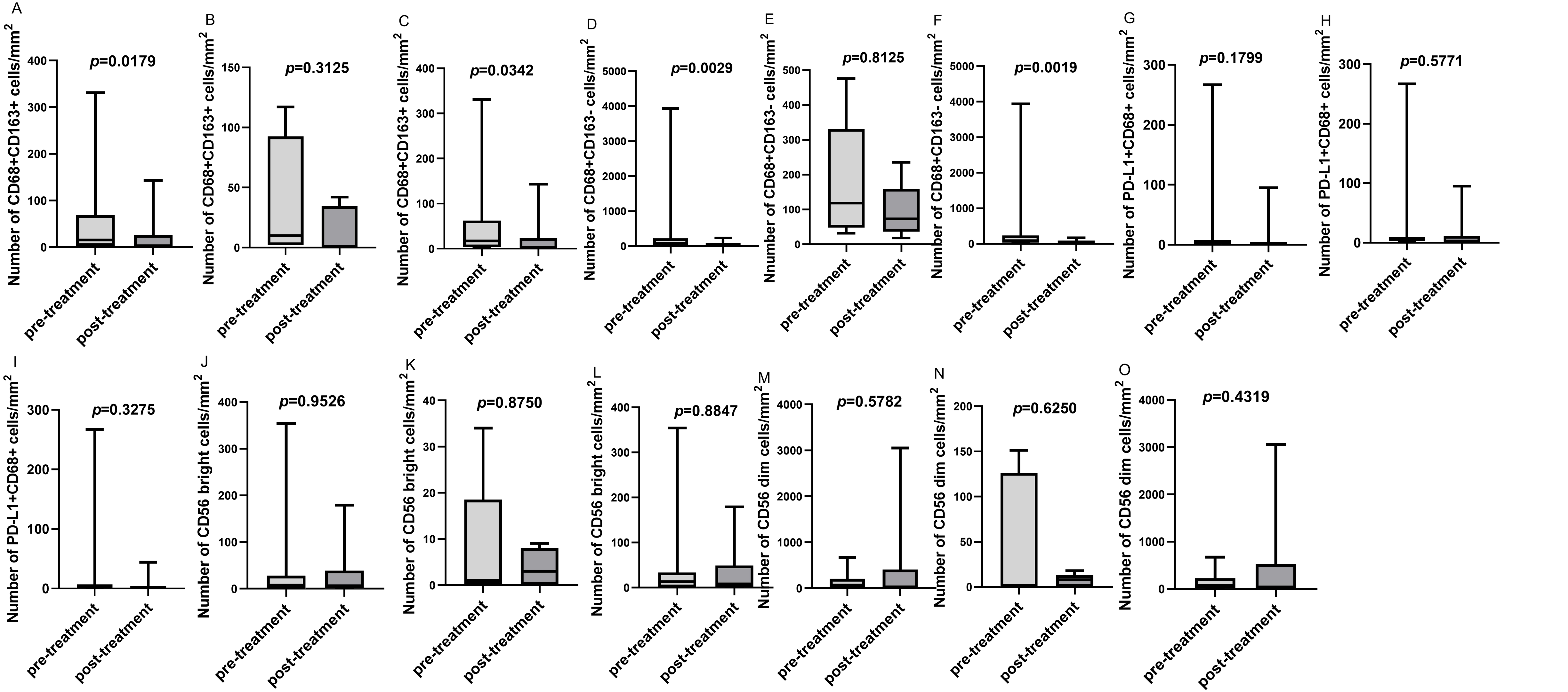

Figure S3: Pre- and post-treatment immune cell infiltration in tumor parenchyma by multiple immunofluorescence staining. Densities of the following cells are compared between pre- and post-treatment samples: (A)infiltrating CD68+CD163+ cells (N=26) , (B) infiltratingCD68+CD163+ cells in patients with TRG 2-3 (N=5) , (C) infiltrating CD68+CD163+ cells in patients with TRG 4-5 (N=21) , (D) infiltrating CD68+CD163- cells (N=26) , (E) infiltrating CD68+CD163- cells in patients with TRG 2-3 (N=5) , (F) infiltrating CD68+CD163- cells in patients with TRG 4-5 (N=21) , (G) infiltrating PD-L1+CD68+ cells (N=26) , (H) infiltrating PD-L1+CD68+ cells in patients with TRG 2-3 (N=5) , (I) infiltrating PD-L1+CD68+ cells in patients with TRG 4-5 (N=21) , (J) infiltrating CD56 bright cells (N=26) , (K) infiltrating CD56 bright cells in patients with TRG 2-3 (N=5) , (L) infiltrating CD56 bright cells in patients with TRG 4-5 (N=21) , (M) infiltrating CD56 dim cells (N=26) , (N) infiltrating CD56 dim cells in patients with TRG 2-3 (N=5) , and (O) infiltrating CD56 dim cells in patients with TRG 4-5 (N=21) .

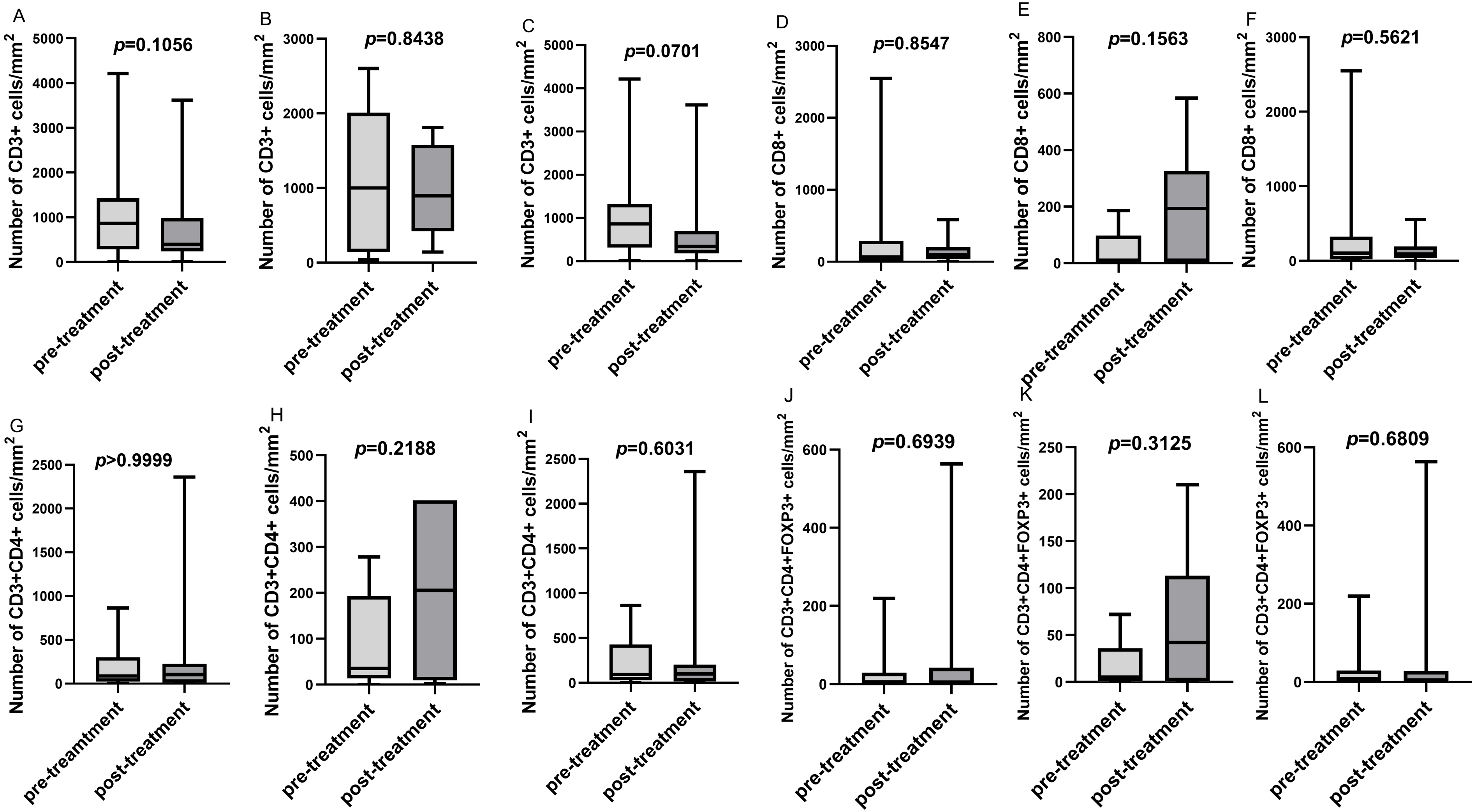

Figure S4: Pre- and post-treatment immune cell infiltration in stroma by multiple immunofluorescence staining. Densities of the following cells are compared between pre- and post-treatment samples: (A)infiltrating CD3+ cells (N=27), (B) infiltrating CD3+ cells in patients with TRG 1-3 (N=6), (C) infiltrating CD3+ cells in patients with TRG 4-5 (N=21), (D) infiltrating CD8+ cells (N=27), (E) infiltrating CD8+ cells in patients with TRG 1-3 (N=6), (F) infiltrating CD8+ cells in patients with TRG 4-5 (N=21), (G) infiltrating CD3+CD4+ cells (N=27), (H) infiltrating CD3+CD4+ cells in patients with TRG 1-3 (N=6), (I) infiltrating CD3+CD4+ cells in patients with TRG 4-5 (N=21), (J) infiltrating CD3+CD4+FOXP3+ cells (N=27), (K) infiltrating CD3+CD4+FOXP3+ cells in patients with TRG 1-3 (N=6), and (L) infiltrating CD3+CD4+FOXP3+ cells in patients with TRG 4-5 (N=21).

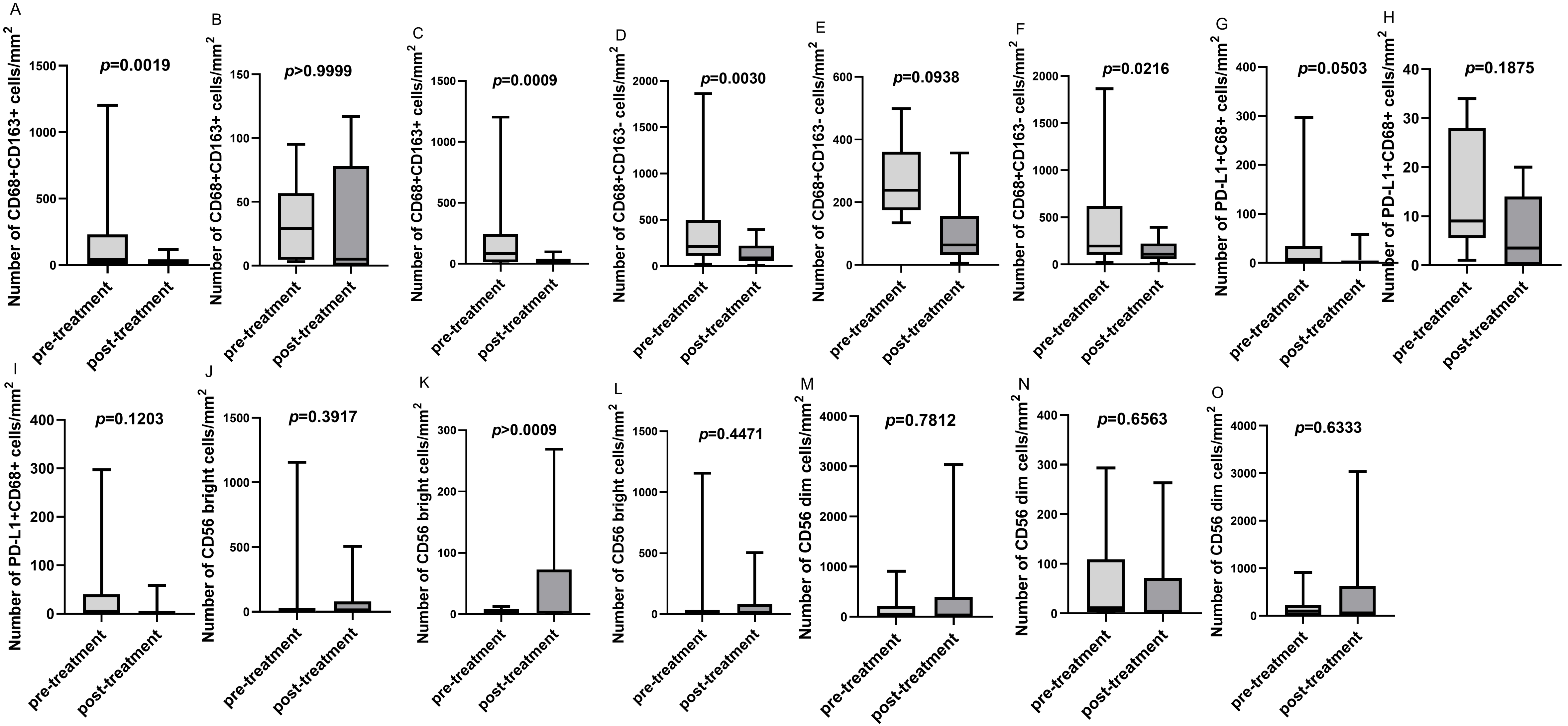

Figure S5: Pre- and post-treatment immune cell infiltration in stroma by multiple immunofluorescence staining. Densities of the following cells are compared between pre- and post-treatment samples: (A)infiltrating CD68+CD163+ cells (N=27), (B) infiltrating CD68+CD163+ cells in patients with TRG 1-3 (N=6), (C) infiltrating CD68+CD163+ cells in patients with TRG 4-5 (N=21), (D) infiltrating CD68+CD163- cells (N=27), (E) infiltrating CD68+CD163- cells in patients with TRG 1-3 (N=6), (F) infiltrating CD68+CD163- cells in patients with TRG 4-5 (N=21), (G) infiltrating PD-L1+CD68+ cells (N=27), (H) infiltrating PD-L1+CD68+ cells in patients with TRG 1-3 (N=6), (I) infiltrating PD-L1+CD68+ cells in patients with TRG 4-5 (N=21), (J) infiltrating CD56 bright cells (N=27), (K) infiltrating CD56 bright cells in patients with TRG 1-3 (N=6), (L) infiltrating CD56 bright cells in patients with TRG 4-5 (N=21), (M) infiltrating CD56 dim cells (N=27), (N) infiltrating CD56 dim cells in patients with TRG 1-3 (N=6), and (O) infiltrating CD56 bright cells in patients with TRG 4-5 (N=21).

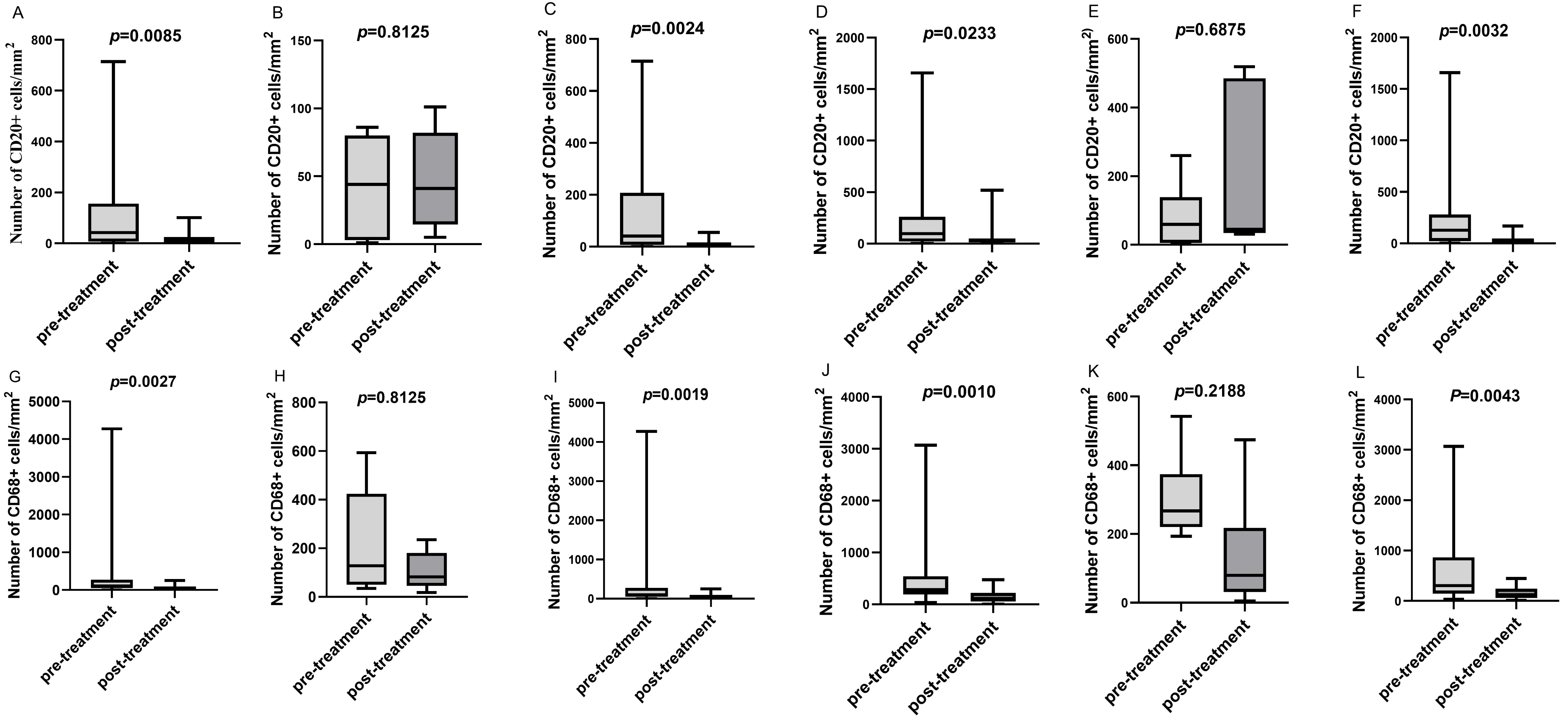

Figure S6: Pre- and post-treatment immune cell infiltration by multiple immunofluorescence staining. Densities of the following cells are compared between pre- and post-treatment samples: (A)infiltrating CD20+ cells in tumor parenchyma (N=26), (B) infiltrating CD20+ cells in tumor parenchyma in patients with TRG 2-3 (N=5), (C) infiltrating CD20+ cells in tumor parenchyma in patients with TRG 4-5 (N=21), (D) infiltrating CD20+ cells in stroma (N=27), (E) infiltrating CD20+ cells in stroma in patients with TRG 1-3 (N=6), (F) infiltrating CD20+ cells in stroma in patients with TRG 4-5 (N=21), (G)infiltrating CD68+ cells in tumor parenchyma (N=26), (H) infiltrating CD68+ cells in tumor parenchyma in patients with TRG 2-3 (N=5), (I) infiltrating CD68+ cells in tumor parenchyma in patients with TRG 4-5 (N=21), (J) infiltrating CD68+ cells in stroma (N=27), (K) infiltrating CD68+ cells in stroma in patients with TRG 1-3 (N=6), and (L) infiltrating CD68+ cells in stroma in patients with TRG 4-5 (N=21).
